# Proceedings of American Dental Association

**Published:** 1882-10

**Authors:** 


### ARTICLE III.

### *American Dental Association.*—(CONTINUED).

THIRD DAY—MORNING.

Dr. J. M. Odell read a paper on diathesis and cachexia and their influence on diseases, and pointed out the necessity of a more thorough knowledge of the family history.

Dr. Atkinson: There is a diffence of opinion as to heredity, and we should take the middle ground.

Dr. Geo. F. Friedrich: Few, if any, medical men understand the *modus operandi* of the medicines they prescribe, and it is nonsense to talk of the molecular affinity of the medicines for the tissue. In a discussion of this kind no facts are brought forward, and we come to no definite conclusions.

Dr. T. L. Buckingham; We use medicine as we do lubricants in machinery. If we put turpentine on a bearing we soon have friction that rapidly wears out the mchine; we do not know how these medicines act, but we know that if we put certain medicines into the machinery we get certain effects. Some act as a whole, some by decomposition. The composition of some substances is nearly like certain articles of food, but like a word, the transposition of whose letters makes an entirely different word.

Dr. Watt: The statement that we do not understand how medicine act is too general. We do not understand how some medicines act, and yet of others. Knowledge is partial. In the treatment of autumnal fevers with arsenious acid, it is administered to interfere with the cause and the root of the disease and kill the morbid tissues. We expect it to kill the morbid tissues and leave the live ones intact. In an anæmic girl, where there is a lack of red corpuscles and, as iron will produce red corpuscles, we should administer it in a form that is most assimilable, or we may give iron by hydrogen and direct the patient to sprinkle it on her bread and butter. It is contented that this form of iron is not assimilable, but I have prescribed it and it has produced the desired result.

Dr. Friedricks; Physicians are supposed to know the cause of a disease before they treat it. Now, what is the cause of fever?

Dr. Watt: That question was settled some time ago, but few people know that it is settled.

Dr. W. O. Kulp, Davenport, Iowa (to Dr. Watt): Perhaps the reason of the iron having the desired effect was because of its being sprinkled on bread and butter.

Dr. Odell: I think Dr. Watt made out a clear case that he did not know any thing about the action of medicines. Dr. Friedrichs is right. There is always a point beyond which we cannot go; we cannot get at the how they operate. Some act by assimilation, others by presence, as Bismuth. Dr. Watt simply stated that arsenic cures certain diseases, but did not give us a glimmering of its *modus operandi.* It is supposed to act on the nervous system, and by a molecular motion cause the elimination of certain substances.

Dr. Watt: We do know that these substances die under the influence of arsenic, and they and the arsenic come out together, thereby giving the system a chance to act by dunging it out, as they say in West Pennsylvania. We know as much about some medicines as we know about anything. We know a plow turns a furrow and pulverizes the soil, yet we cannot explain how it is done.

Dr. Odell: About all we know about medicine is at first empirical. Science is simplya classification of empirical knowledge. Some one discovers a result, and we go to work to discover how it is produced, but we have never yet found out how this is done except in the case of the oxygenation of the blood.

Dr. Atkinson: We talk of stimulant and tonics; how do they act? The body is made up of molecules; any impact that enters into a molecule and becomes part of it is a tonic, and any impact that accelerates molecular action is a stimulant; every atom has a static and a dynamic side. It is difficult to imagine the creation of atoms; any substance that can act as a molecule will, according to the condition of the molecule, act as a food or poison.

Dr. Friedrichs: Does any one else believe that?

Prof. Mayr: Dr. Watt says that arsenic kills organisms. In my laboratory I had too bottles, one containing a solution of arsenious acid, and the other arsenite of soda, and the liquid in both bottles became covered with mould. I consider arsenic a stimulant of the nervous system.

Dr. E. T. Darby: We do not know how arsenic acts on a pulp. Opinions differ. Dr. Flagg made some experiments with arsenic. He took one twenty-fifth of a grain on a piece of cotton and applied it to pulp and devitalized it. He then applied the same arsenic to another pulp with the same result, and so on until he had devitalized ten pulps. He then applied it to the web of a frog's foot and killed it. The question is, how was it possible to acomplish those results?

Dr. T. L. Buckingham: I think I know something about that frog. We found what we supposed to be arsenic in all parts of it. We do know that a very small quantity of arsenic will have a good deal of effect, but this does not prove that it may not be absorbed. I do not know how it destroys pulps. When arsenic is applied to tumor, it attack the weakest tissues first, which makes a cushion that retards its action. We cannot say why sulphur and oxygen combine. There are law that regulate them like letters which can be put together, but must be put in such shape as to form words. We know nothing about matter; we only know it by its properties, not by its substance.

Dr. Watt: Does not arsenious acid coagulate albumen?

Dr. Buckingham: Yes.

Prof. Mayr: Has anybody ever noticed that arsenic coagulates album?

Dr. Buckingham: We know that immersing seeds in a solution of arsenic will prevent their germinating, and after it has been applied to the pulp, and that organ is apparently dead, the arsenic may be removed by syringing with water and the pulp restored to life. It paralyzes, it acts in the same way as cold.

Dr. George R. Thomas, Detroit: Can the arsenic be removed and the pulp be restored to life again?

Dr. Buckingham: There is a mere suspension of vitality.

Dr. W. W. Allport. Chicago: It is difficult to explain why remedies act; we only know they do act. We know not how we live; Dr. Flagg's experiments were not thoroughly understood; he wished to prove that it destroyed vitality, was not due to absorption, but it acted as an irri tant, and he showed that it did not exist in the pulp save at the point of contact· The irritation of the pulp caused con gestion and strangulation at the apex. I have found that when the pulp has not been wounded that it takes the least quantity of arsenic to destroy it, but when it has been wounded it takes a larger quantity ; this is due to a loss of blood. In other cases I have introduced a quantity of arsenic, and in a few days afterward a portion of the pulp has sloughed off and the rest of the organ remained alive for years.

Dr. J. N. Crouse, chairman of the committee appointed to draft resolutions in memory of the late Dr. M. S. Dean, of Chicago, reported a paper, which was ordered spread on the memorial page of the transactions of the association. It states that in the sad bereavement they had lost the companionship of one whe was original and progessive in thought, though calm and conservative in action, and just and charitable in expressing his convictions, posessing keen perceptions, delicate sensibilities, and refined tastes ; he was pure, warmheated, and sympathetic in nature, genial, humorous, witty, and at times even boyish and exuberant in spirits. With these attributes governing his life he enjoyed to an unusual degree the confidence and affection of his associates. They had lost a scholarly, cultivated, and generous friend, who has filled with honor and great satisfaction every responsible position at their disposal. His brillant intellectual attainments, earnest, honest, and modest bearing, made him not less dear to their scientific friends abroad than to themselves. The heartfelt sympathies of the association were accorded to the bereaved family.

Dr. W. C. Barrett, Chairman of Section 7, on physiology and etiology, reported the great impotance of this new section, and in reference to a suggestion in the President's address the following was read : That the sum of $200 be set apart to be adjudged by a special committee of three membes to be appointed by the president of the Association to the author of the best paper on the etiology of dental caries, the paper to be based upon strictly original investgation, and to be presented to this Association in the report of Section 7. Such committee shall prescribe the conditions upon which essays shall be presented, and in case of two or more papers of equal claim to merit they may in their discretion divide the sum so set apart and award a portion to each essayist. Unless papers of sufficiently original merit be submitted, the committee may decline to award the prize, and hold it over until it has been fairly earned. The competitors for the prize essay need not be members of this Association, but the profession of the world is invited to the consideration of the subject.

Dr. Barrett also read a paper on "The Origin of Nervous Force." It explained the origin of nervous force, he said, and endeavored to prove, that it was a correlation of original force—force as it is in general and in its essence. In that sense it was a continuation or a complement of original forces, such as heat, electricity, and like these, it was derived from the molecular action of chemical affinitee.

He mentioned an example of his position which he had given recently at a meeting of a State Dental Association. He had exposed the inside organs of a dog—the heart had been bared to view. Soon the beating of the heart ceased, as though life were extinct. Some friends of his had retired and declared that his experiment was a failure. But he said it was not, and applied a shock of electricity, at the same time infusing the proper amount of heat. The result was that the heart began to beat as regularly as before the operation commenced. The scientific research of the day was for the conservation of nervous force.

(At the conclusion of Dr. Barrett's paper Dr. Buckingham made some remarks, but owning to the noise and confusion they were inaunible.—*Rep*).

THIRD DAY—AFTERNOON.

After deciding to meet at Niagara next year the Association went into an election of officers for the ensuing year with the following result.

Dr. W. H. Goddard, of Louisville, President; Dr. Geo. J. Freidrichs, of New Orleans, First Vice President; Dr. A. W. Harlan, of Chicago, Corresponding Secretary; Dr. Geo. H. Cushing, of Chicago, Recording Secretary; Dr. Ceo. W. Keely, of Xenia, Ohio, Treasurer; Executive Committee, Dr. F. M. Odell, T. T. Moore, S. G. Perry, C. N. Pierce, W. H. Morgan, F. H. Rehwinkle, Dr. J. N. Course, Dr. A. M. Dudley, G. H. H. Field.

### Fourth Day—Morning.

During the morning session the matter of appropriating a certain amount for best original essays on Dental Caries was considered, and it was decided to appropriate $200 for that purpose. Dr. Buckingham, of Philadelphia, explained the substance of a paper on artificial dentistry. He referred to the importance of the use of celluloid in the manufacture of teeth. Celluloid, by the application of heat from 290° to 310° well regulated by a thermoneter, might be put into such a condition as to be easily pliant to any form. He deprecated the fact that so many dentists were careless in the manufacture of teeth. They made a set of teeth, for instance, for an old man as though he was scarcely twenty-one—thus the naturalness of teeth was not at all preserved. Celluloid would not only be plastic, but would receive any color. Dr. Dorrance, of Ann Arbor, Mich., read a paper on the ill results of vegetable bases. He also exhibited a new blowpipe, and specified a new compound for solder made of silver zinc, and copper. Upon motion of Dr. Barett, it was resolved to declare the sense of the meeting to be that the new blowpipe be manufactured by the inventor for the benefit of dentists.

In the afternoon President elect Goddard was installed as the presiding officer. He made a strong address in taking the chair, which was loudly applauded. He closed it by saying: "And I assume the gravel feeling a consciousness of success." Some time was spent by seveal members expressing their sentiments in regard to the deaths of Dr. Hawxhurst and Dr. Dean.

Dr. Taft read a paper on dental literature. The United States, the Doctor said had a larger number of dental journals than the world besides. The dental journals of the United States contain each month 425 pages 5,100 per year The British Journals contained 1,692 pages per year, the French 984, making in all, 4,824 pages. According to this estimate the periodical literaure of the United States exceeded that of all other countries by 276 pages.

Dr. J. W. Crouse, read a paper on Dental Education. He took the position that, on account of the award of diplomas, college education had been deteriorated. The paper provoked considerable discussion. Dr. Atkinson, of New York said the time of graduation had nothing to do with the two, three, or even ten years, made little difference as to his knowledge unless in that time he had acquired it. Dental education had grown, but, not like ripe fruit, it had become abundant, but its fruits green. Only by honesty would the dental profersion attain to a decent standard of education. Dr. Pierce insisted on the importance of a State Board of Examiners being appointed, taking the duty of examination entirely out of the hands of a faculty. Dr. Barrett, of Buffalo, favored this view, but qualified his position by saying that in the present condition of things it was very important to require a course of a certain number of years from the colleges, otherwise the abuse of granting diplomas would be on the increase. Dr. J. Taft said it was easy enough to describe an evil, but to apply a suitable remedy was a difficult matter. Dr. Morgan, of Nashville, said it was not true that responsible colleges were wont to grant diglomas where they were not deserved. He did not believe in a State Board of Examiners, because a professor in the college, who taught the student every day, was the only competent man to examine him. He was diametrically opposed to a union of medical and dental colleges. Dental students ought to be educated in dental colleges by dental professors. Dr. Allport, of Chicago, averred that it was useless to build up dental science on any other foundation than that of medicine. The greatest quacks were the graduates of dental colleges. Dr. Hunter took the position that a good dental education must first be acquired in a regular medical college. Drs. Barrett, Chas. Butler, and Field, were appointe a committee to make arrangements for the accommodation of members at next place of meeting at Niagara Falls, to act in concert with the first division of the Executive Committee. Dr. Darby, of Pennsylvania, and Dr. Harlan of Chicago, were appointed the committee on publication.—*Cin-cinnati Lancet aud Clinic.*

